# Comparative Study of Bilateral Uterine Artery Ligation Versus B-Lynch Suture in Primary Postpartum Hemorrhage Following Lower Segment Cesarean Section: A Prospective Single-Center Analysis

**DOI:** 10.7759/cureus.86778

**Published:** 2025-06-26

**Authors:** Ranjana Jha, Ashutosh K Jha, Meenakshi Singh

**Affiliations:** 1 Department of Obstetrics and Gynaecology, Lord Buddha Koshi Medical College, Gamhariya, IND; 2 Department of Anaesthesia, Katihar Medical College, Katihar, IND; 3 Department of Obstetrics and Gynaecology, Lady Hardinge Medical College, Delhi, IND

**Keywords:** b-lynch suture, lower segment caesarean section (lscs), patient follow-up, s: postpartum hemorrhage, uterine artery ligation

## Abstract

Background

Primary postpartum hemorrhage (PPH) remains a significant challenge in obstetric practice, contributing to maternal morbidity and mortality worldwide. Characterized by excessive bleeding within the first 24 hours following childbirth, PPH is a leading cause of maternal death. Uterine atony, inadequate contraction of the uterine muscle, accounts for the majority of PPH cases, particularly following lower segment cesarean section (LSCS). Bilateral uterine artery ligation (BUAL) and B-Lynch sutures have emerged as surgical interventions for managing PPH secondary to uterine atony during LSCS. Despite their demonstrated efficacy, comparative studies are needed to determine the optimal approach.

Methods

This prospective, comparative study was conducted among women experiencing primary postpartum hemorrhage (PPH) due to uterine atony following lower segment cesarean section (LSCS) over two years at a tertiary care hospital in India. A total sample size of 86 participants (43 per group) was determined using a convenient sampling technique within the defined study period. Participants were randomized into two groups: the BUAL group and the B-Lynch suture group. The primary outcome was intraoperative blood loss (ml). Statistical analysis was conducted using IBM Corp. Released 2013. IBM SPSS Statistics for Windows, Version 22.0. Armonk, NY: IBM Corp., with significance set at p < 0.05.

Results

The mean age of study participants was 28.6 years (± 4.2) in the BUAL group and 29.1 years (± 4.6) in the B-Lynch suture group, demonstrating no significant difference (p = 0.563). Furthermore, the length of hospital stay was comparable between the BUAL and B-Lynch suture groups (p = 0.667). Maternal satisfaction, indicated by the proportion of satisfied participants, was high in both groups, with no significant difference observed (p = 0.682). Subgroup analyses based on the mode of delivery and severity of uterine atony within the BUAL and B-Lynch suture groups showed no significant differences in intraoperative blood loss (p-values ranging from 0.491 to 0.802).

Conclusion

In conclusion, both BUAL and B-Lynch sutures demonstrate comparable efficacy and safety in managing primary postpartum hemorrhage (PPH) due to uterine atony following lower segment cesarean section (LSCS). Further large-scale studies are warranted to validate these findings and inform evidence-based clinical practice in managing PPH during LSCS.

## Introduction

Primary postpartum hemorrhage (PPH) continues to pose a substantial challenge in obstetric practice, accounting for a significant proportion of maternal morbidity and mortality worldwide [1]. Defined as excessive bleeding (> 500 ml) within the first 24 hours following childbirth, PPH is a leading cause of maternal mortality, responsible for approximately one-quarter of all maternal deaths globally [2].

Primary PPH significantly contributes to maternal morbidity and mortality across the globe. The World Health Organization (WHO) estimates that PPH causes approximately 60,000 maternal deaths each year, highlighting its impact on maternal health [3]. The condition is particularly prevalent in low-resource settings where access to timely and adequate healthcare is limited, exacerbating the risk of severe outcomes [4].

PPH is particularly common after LSCS due to the disruption of the uterine muscle and blood vessels during the surgical procedure. The lower uterine segment is less contractile compared to the upper segment, making it more prone to atony and subsequent hemorrhage [5]. This increased risk underscores the importance of vigilant monitoring and prompt management in women undergoing LSCS.

Among the myriad causes of PPH, uterine atony, characterized by inadequate contraction of the uterine muscle following delivery, is the predominant etiology, accounting for 70-80% of cases [6]. Uterine atony can result from factors such as overdistension of the uterus, prolonged labor, chorioamnionitis, and the use of certain anesthetics [7]. Exploring effective interventions to manage uterine atony is crucial, as failure to promptly address this condition can lead to severe hemorrhage and adverse maternal outcomes.

Despite advances in obstetric care, including the widespread use of uterotonic agents and improved surgical techniques, a subset of women undergoing LSCS still experience primary PPH refractory to conventional management strategies [8]. In such cases, timely and effective surgical interventions are imperative to prevent adverse maternal outcomes [8].

Bilateral uterine artery ligation (BUAL) and B-Lynch sutures have emerged as viable surgical options for managing PPH secondary to uterine atony during LSCS. BUAL involves the occlusion of the uterine arteries bilaterally, thereby reducing blood flow to the uterus and potentially arresting hemorrhage [7,8]. In contrast, the B-Lynch suture, named after its inventor, involves the placement of a horizontal compression suture around the uterine body, effectively creating mechanical hemostasis [9].

While both BUAL and B-Lynch sutures have demonstrated efficacy in controlling PPH, particularly in cases of uterine atony, there remains a lack of consensus regarding the optimal surgical approach in this clinical scenario [10,11]. Existing literature comprises primarily case series, retrospective studies, and small-scale randomized controlled trials (RCTs), with inconsistent findings regarding the comparative effectiveness and safety of these interventions [10-12]. Consequently, there is a compelling need for robust comparative studies to elucidate the relative merits of BUAL versus B-Lynch suture in the management of primary PPH following LSCS due to uterine atony.

Therefore, this study aimed to compare the efficacy, safety, and maternal outcomes associated with BUAL versus B-Lynch suture in the management of primary PPH during LSCS.

## Materials and methods

Study design and study setting

This prospective, comparative study was conducted among women with primary postpartum hemorrhage (PPH) due to uterine atony following lower segment cesarean section (LSCS) during a period of two years from July 2021 to June 2023 in the Department of Obstetrics and Gynecology at a tertiary care hospital, India. Informed consent was obtained from all participants before enrollment.

Study population

The study population consisted of women undergoing elective or emergency LSCS at the study center who were diagnosed with primary PPH due to uterine atony. Inclusion criteria encompassed women aged 18 years or older with singleton pregnancies. Exclusion criteria included contraindications to BUAL or B-Lynch suture, prior uterine surgery, known coagulopathy, and inability to provide informed consent. Using a convenient sampling technique, a total sample size of 86 participants (43 in each group) in the study within defined a study period.

Surgical interventions

Participants were allocated into two groups: the BUAL group and the B-Lynch suture group. The surgical procedures were performed by experienced obstetric surgeons trained in both the BUAL and B-Lynch techniques. For participants in the BUAL group, after standard preoperative preparation and confirmation of uterine atony as the cause of primary postpartum hemorrhage (PPH), a transverse abdominal incision was made under appropriate anesthesia. The uterine arteries were identified bilaterally at their origin from the internal iliac arteries. Using either laparoscopic or open surgical techniques based on surgeon preference, the uterine arteries were carefully dissected and ligated using non-absorbable suture material. Hemostasis was confirmed, and the surgical site was inspected for any bleeding before the closure of the abdominal incision. Additional measures such as administration of uterotonic agents, blood transfusion, or hysterectomy were undertaken as deemed necessary to control hemorrhage. Postoperative care was standardized across both groups, including monitoring for complications, administration of antibiotics, pain management, and counseling on postpartum recovery and contraception. Conversely, participants in the B-Lynch suture group underwent similar preoperative preparation and anesthesia. Following confirmation of uterine atony, the B-Lynch compression suture technique was employed. A series of horizontal compression sutures were placed around the uterine body using non-absorbable suture material in a U-shaped fashion to achieve hemostasis. Care was taken to avoid injury to adjacent structures during suture placement. Hemostasis was confirmed, and the surgical field was inspected for any bleeding before the closure of the abdominal incision. Additional measures such as administration of uterotonic agents, blood transfusion, or hysterectomy were undertaken as deemed necessary to control hemorrhage. Postoperative care was standardized across both groups, including monitoring for complications, administration of antibiotics, pain management, and counseling on postpartum recovery and contraception.

Outcome measures

The primary outcome measure was intraoperative blood loss, quantified in milliliters. Secondary outcome measures included the requirement for additional interventions to control hemorrhage (e.g., uterotonic agents, blood transfusion, hysterectomy), incidence of postoperative complications (infection, thromboembolism), length of hospital stay, and maternal mortality.

Statistical analysis

Statistical analysis was performed using appropriate parametric or non-parametric tests, depending on the distribution of data, using IBM Corp. Released 2013. IBM SPSS Statistics for Windows, Version 22.0. Armonk, NY: IBM Corp. Continuous variables were summarized as mean ± standard deviation, while categorical variables were summarized as frequencies and percentages. Between-group comparisons were conducted using independent t-tests for continuous variables and chi-square tests for categorical variables. A two-tailed p-value of < 0.05 was considered statistically significant.

Ethical considerations

The study protocol was reviewed and approved by the institutional ethical and review board (approval number: LBKMC/IERB/2021/2/2, date: 05/02/2021), and informed consent was obtained from all participants. Measures were taken to ensure patient confidentiality and data security throughout the study period.

## Results

In the present study, 43 participants were enrolled and allocated into either the bilateral uterine artery ligation (BUAL) or B-lynch suture group, contributing to the comparative analysis of these interventions in managing primary postpartum hemorrhage during lower-segment cesarean section. The mean age of participants was 28.6 years (± 4.2) in the BUAL group and 29.1 years (± 4.6) in the B-Lynch suture group, demonstrating no significant difference (p = 0.563). Regarding parity, 21 (48.8%) participants in the BUAL group were primiparous, compared to 19 (44.2%) in the B-Lynch suture group, with 22 (51.2%) and 24 (55.8%) being multiparous, respectively (p = 0.665). The mean gestational age at cesarean section was 38.4 weeks (± 1.2) in the BUAL group and 38.6 weeks (± 1.4) in the B-Lynch suture group, with no statistically significant distinction (p = 0.419). The distribution of emergency versus elective lower segment cesarean section (LSCS) was similar between the groups (p = 0.641), as was the categorization of uterine atony into mild and severe cases (p = 0.641). Moreover, the prevalence of comorbidities such as hypertension (18.6% (n=8) vs. 16.3% (n=17), p = 0.789) and diabetes (11.6% (n=5) vs. 14.0% (n=6), p = 0.746) did not significantly differ. Most participants received adequate antenatal care, with 35 (81.4%) in the BUAL group and 33 (88.4%) in the B-Lynch suture group having four or more visits, though the disparity was not statistically significant (p = 0.366). The occurrence of moderate anemia (hemoglobin < 10 g/dL) was comparable between the groups, with six (14.0%) in the BUAL group and four (9.3%) in the B-Lynch suture group (p = 0.501) (Table 1).

**Table 1:** Baseline characteristics of the study participants. LSCS: Lower segment cesarean section, BUAL: Bilateral uterine artery ligation, Statistically significant for p-value <0.05

Characteristic	BUAL Group (n=43)	B-Lynch Suture Group (n=43)	p-value
	Frequency (%)/ Mean ± SD		
Age (years)	28.6 ± 4.2	29.1 ± 4.6	0.563
Parity			
Primiparous	21 (48.8%)	19 (44.2%)	0.665
Multiparous	22 (51.2%)	24 (55.8%)	
Gestational Age (weeks)	38.4 ± 1.2	38.6 ± 1.4	0.419
Indication for LSCS			
Emergency	28 (65.1%)	26 (60.5%)	0.641
Elective	15 (34.9%)	17 (39.5%)	
Uterine Atony			
Mild	15 (34.9%)	17 (39.5%)	0.641
Severe	28 (65.1%)	26 (60.5%)	
Comorbidities			
Hypertension	8 (18.6%)	7 (16.3%)	0.789
Diabetes	5 (11.6%)	6 (14.0%)	0.746
Antenatal Care Visits			
4 or more	35 (81.4%)	38 (88.4%)	0.366
Less than 4	8 (18.6%)	5 (11.6%)	
Anemia (Hemoglobin < 10 g/dL)	6 (14.0%)	4 (9.3%)	0.501

In our study, there were no significant differences observed in intraoperative blood loss (p = 0.703) or hemoglobin change (p = 0.279) between the groups. Additionally, the need for additional interventions, including the administration of uterotonic agents (p = 0.693), blood transfusion (p = 0.556), and hysterectomy (p = 0.314), did not significantly differ between the groups. Rates of postoperative complications, such as infection (p = 0.644), thromboembolism (p = 0.682), wound dehiscence (p = 0.987), and urinary tract infection (p = 0.693), were similar between the two groups. There were no cases of maternal mortality in either group, indicating the effectiveness of both surgical techniques in managing severe postpartum hemorrhage and preventing fatal outcomes. Furthermore, the length of hospital stay was comparable between the BUAL and B-Lynch suture groups (p = 0.667). Maternal satisfaction, indicated by the proportion of satisfied participants, was high in both groups, with no significant difference observed (p = 0.682) (Table 2).

**Table 2:** Comparison of outcome measures among two groups. BUAL: Bilateral uterine artery ligation, Statistically significant for p-value <0.05

Outcome Measure	BUAL Group (n=43)	B-Lynch Suture Group (n=43)	p-value
	Frequency (%)/ Mean ± SD		
Intraoperative Blood Loss (ml)	1277.9 ± 257.6	1299.7 ± 271.6	0.703
Hemoglobin Change (g/dL)	-2.1 ± 0.9	-2.3 ± 0.8	0.279
Additional Interventions Required	5 (11.6%)	4 (9.3%)	0.724
Additional Uterotonic Agents	4 (9.3%)	3 (7.0%)	0.693
Blood Transfusion	2 (4.7%)	1 (2.3%)	0.556
Hysterectomy	1 (2.3%)	0 (0.0%)	0.314
Postoperative Complications	7 (16.3%)	6 (14.0%)	0.763
Infection	3 (7.0%)	2 (4.7%)	0.644
Thromboembolism	2 (4.7%)	3 (7.0%)	0.682
Wound Dehiscence	1 (2.3%)	1 (2.3%)	0.987
Urinary Tract Infection	4 (9.3%)	3 (7.0%)	0.693
Length of Hospital Stay (days)	4.8 ± 2.2	4.6 ± 2.1	0.667
Maternal Satisfaction	40 (93.0%)	41 (95.3%)	0.682

We did subgroup analyses based on the mode of delivery and severity of uterine atony within the BUAL and B-Lynch suture groups. In the emergency mode of delivery subgroup, mean intraoperative blood loss was 1285.4 ml (± 257.4) in the BUAL group and 1301.2 ml (± 281.2) in the B-Lynch suture group, with no significant difference observed (p = 0.735). Similarly, in the elective mode of delivery subgroup, mean blood loss was 1267.1 ml (± 250.3) and 1285.6 ml (± 274.6) in the BUAL and B-Lynch suture groups, respectively, with no significant difference noted (p = 0.744). Regarding uterine atony severity, participants with mild atony had mean blood loss of 1261.6 ml (± 256.5) in the BUAL group and 1292.5 ml (± 274.3) in the B-Lynch suture group, showing no significant difference (p = 0.592). In the severe atony subgroup, mean blood loss was 1302.4 ml (± 269.6) and 1320.3 ml (± 285.4) in the BUAL and B-Lynch suture groups, respectively, with no significant difference observed (p = 0.491) (Table 3).

**Table 3:** Subgroup analysis of outcome measures among two groups. BUAL: Bilateral uterine artery ligation, Statistically significant for p-value <0.05

Subgroup	BUAL Group (n=43)	B-Lynch Suture Group (n=43)	p-value
	Mean ± SD		
Mode of Delivery			
Emergency (n=54)	1285.4 ± 257.4	1301.2 ± 281.2	0.735
Elective (n=32)	1267.1 ± 250.3	1285.6 ± 274.6	0.744
p-value	0.736	0.802	
Uterine Atony			
Mild (n=32)	1261.6 ± 256.5	1292.5 ± 274.3	0.592
Severe (n=54)	1302.4 ± 269.6	1320.3 ± 285.4	0.658
p-value	0.491	0.659	

## Discussion

Postpartum hemorrhage (PPH) remains a significant challenge in obstetric care, particularly following cesarean section deliveries, where the risk of uterine atony is heightened. In our study, we sought to elucidate the comparative effectiveness of bilateral uterine artery ligation (BUAL) versus B-Lynch suture in managing primary PPH attributed to uterine atony following lower segment cesarean section (LSCS). Our findings contribute to the growing body of knowledge surrounding the optimal management of PPH and provide insights into the clinical utility of these interventions.

The mean age of participants was 28.6 years (± 4.2) in the BUAL group and 29.1 years (± 4.6) in the B-Lynch suture group, demonstrating no significant difference (p = 0.563). A similar mean age was observed in the studies by Gora et al. and Thawal et al. [13,14].

Regarding parity, 48.8% of participants in the BUAL group were primiparous, compared to 44.2% in the B-Lynch suture group, with 51.2% and 55.8% being multiparous, respectively (p = 0.665), which is similar to the studies by Kumar et al., and Vidyadhar et al. [15,16].

The mean gestational age at cesarean section was 38.4 weeks (± 1.2) in the BUAL group and 38.6 weeks (± 1.4) in the B-Lynch suture group, with no statistically significant distinction (p = 0.419). Similar gestational age was observed in the studies by Kebede et al., and Tongde et al. [17,18].

Our observation that both BUAL and B-Lynch suture interventions effectively controlled intraoperative blood loss without a significant difference between the two groups underscores the importance of these techniques in achieving hemostasis (BUAL: mean 1277.9 ml ± 257.6, B-Lynch suture: mean 1299.7 ml ± 271.6, p = 0.703). The efficacy of BUAL lies in its ability to disrupt blood flow to the uterus by ligating the uterine arteries bilaterally, thereby reducing uterine perfusion and hemorrhage. Similarly, the B-Lynch suture, a mechanical compression technique, functions by exerting pressure on the uterine body to achieve hemostasis, thus arresting bleeding secondary to uterine atony. The comparable outcomes observed in our study are consistent with previous studies by Wankhede et al., Gadappa et al., Das et al., and Devendra et al., highlighting the effectiveness of both interventions in controlling blood loss during cesarean section procedures [19-22]. Conversely, Abdel-Fatah et al. found significantly lower mean blood loss with BUAL (770.19 mL) compared to B-Lynch suture (785.91 mL) [23]. Notably, Puangsricharoen et al. reported substantially higher mean blood loss values for both interventions, with 2133 mL for BUAL and 2984 mL for B-Lynch suture [24]. 

The scientific rationale behind the effectiveness of both interventions lies in their mechanistic pathways. BUAL reduces uterine perfusion by ligating the uterine arteries, thereby decreasing blood flow to the uterus and mitigating hemorrhage. This technique leverages the anatomical positioning of the uterine arteries to achieve rapid and effective hemostasis. The reduction in blood flow results in decreased uterine perfusion, which helps in controlling the bleeding [6,8]. On the other hand, the B-Lynch suture achieves hemostasis through mechanical compression. By applying pressure around the uterine body, the suture compresses the uterine blood vessels, which helps in controlling bleeding. This method is particularly effective in cases where uterine atony is the primary cause of PPH, as it directly addresses the lack of uterine muscle contraction by providing external support to maintain hemostasis. The compression created by the B-Lynch suture promotes the formation of blood clots within the uterine vasculature, thus stopping the hemorrhage [10,11].

Furthermore, our study found similar rates of additional interventions required, such as the administration of uterotonic agents, blood transfusion, and hysterectomy, between the BUAL and B-Lynch suture groups (additional interventions: BUAL 11.6% vs. B-Lynch suture 9.3%, p = 0.724). This suggests that both interventions adequately addressed the underlying cause of PPH and minimized the need for further interventions to achieve hemostasis. These findings lie in the mechanisms of action of BUAL and B-Lynch sutures, which target the underlying etiology of uterine atony, thereby mitigating the need for additional therapeutic measures [25]. In contrast, Dohbit et al. reported a 66.7% success rate for BUAL and 100% for B-Lynch suture in their study [26]. 

The absence of significant differences in postoperative complication rates, including infection, thromboembolism, wound dehiscence, and urinary tract infection, between the two intervention groups underscores the safety and feasibility of both BUAL and B-Lynch suture techniques in the management of PPH. While the exact mechanisms underlying these outcomes may vary, both interventions aim to achieve hemostasis while minimizing the risk of adverse events, thereby ensuring optimal patient outcomes and safety [27].

Moreover, our study revealed comparable lengths of hospital stay between the BUAL and B-Lynch suture groups, indicating similar recovery times following the two interventions (BUAL: mean 4.8 days ± 2.2, B-Lynch suture: mean 4.6 days ± 2.1, p = 0.667). This suggests that both techniques are associated with comparable postoperative recovery trajectories, allowing for timely discharge and postoperative care. A similar period of hospital stay was observed in the study by Nayek et al. [28]. In a study by Warade et al., 12% of participants had hospital stays longer than five days [29].

So, it suggests that both surgical techniques are equally effective in managing blood loss during LSCS complicated by uterine atony. This finding supports the flexibility in surgical choice based on the surgeon's expertise and available resources, rather than a strict preference for one technique over the other. However, it's important to consider potential factors not accounted for in our analysis that might influence outcomes. These could include variations in surgical skill levels, differences in institutional protocols for managing PPH, patient-specific factors such as body mass index (BMI), preexisting medical conditions, and the timing and dosage of administered uterotonic agents. 

Limitations

A few limitations are acknowledged in this study. First, the sample size was relatively small, potentially limiting the generalizability of the findings. Additionally, the study was conducted at a single tertiary care center, which may introduce institutional bias and limit the extrapolation of results to other settings. Furthermore, the study design, although prospective and comparative, may still be susceptible to confounding factors despite attempts to control for them. Moreover, the short-term follow-up period precludes the assessment of long-term outcomes and complications.

Future goals

These limitations highlight the need for larger multicenter studies with longer follow-up periods to provide more robust evidence regarding the comparative effectiveness of BUAL and B-Lynch suture in managing primary PPH during LSCS. Future research will aim to include a more diverse patient population from multiple centers to enhance the generalizability of the findings. Additionally, extending the follow-up period will allow for the evaluation of long-term outcomes and potential complications, providing a more comprehensive understanding of the benefits and risks associated with each surgical technique. By addressing these limitations, future studies will contribute to the development of evidence-based guidelines for the management of primary postpartum hemorrhage.

## Conclusions

The finding of comparable intraoperative blood loss between the BUAL and B-Lynch suture groups has significant implications for evidence-based decision-making in the management of postpartum hemorrhage during lower-segment cesarean section (LSCS). This equivalence suggests that both techniques are similarly effective in controlling blood loss, offering clinicians flexibility in choosing the most appropriate intervention based on individual patient circumstances and surgical expertise. By demonstrating that both BUAL and B-Lynch sutures are effective in reducing intraoperative blood loss, the study supports the inclusion of both techniques in clinical guidelines for managing primary postpartum hemorrhage. Clinicians can consider other factors such as the patient's medical history, the availability of surgical expertise, and institutional resources when selecting the optimal intervention. Furthermore, this finding underscores the importance of training obstetric surgeons in both techniques to ensure they are equipped to manage postpartum hemorrhage effectively, regardless of the chosen method. In conclusion, the comparable intraoperative blood loss between the groups reinforces the utility of both BUAL and B-Lynch sutures as viable options in the surgical management of primary postpartum hemorrhage during LSCS, contributing to informed and flexible clinical decision-making.
